# Supplementary material for: Migrant mothers’ experiences of postnatal depression in the UK
Source: PLoS One. 2026 May 6;21(5):e0347584. doi: 10.1371/journal.pone.0347584 (PMC13148705; doi:10.1371/journal.pone.0347584)
Supplement: S1 File — (DOCX) [file pone.0347584.s001.docx]

**Project Timeline:**

| **PROCESS** | **TIME** |
| --- | --- |
| MRP Proposal Hand in | February 2024 |
| Registration Viva and oral research examination | March 2024 |
| UH ethics submission | April 2024 |
| Start systematic literature review, finalise interview schedule with consultation and recruitment strategy | May 2024 |
| Continue systematic literature review, start recruitment | June 2024 |
| Start data collection via interviews and transcriptions | July 2024 |
| Finalise introduction and systematic literature review | September 2024 |
| Start qualitative analysis | December 2024 |
| Write methodology section | March 2025 |
| Write results and discussion | April 2025 |
| Final draft submission | June 2025 |
| Viva Voce | July 2025 |
| Thesis amendments and drafting journal paper | July-September 2025 |
| Dissemination | September 2025 onwards |
