# Supplementary material for: Migrant mothers’ experiences of postnatal depression in the UK
Source: PLoS One. 2026 May 6;21(5):e0347584. doi: 10.1371/journal.pone.0347584 (PMC13148705; doi:10.1371/journal.pone.0347584)
Supplement: S2 File — (DOCX) [file pone.0347584.s002.docx]

**Screening call guide**

**Approximately 15-30 minutes**

-Introductions

-Share reason for topic of research

-Share rationale for screening call: determining eligibility, share details of the study procedure and opportunity to discuss any queries or concerns regarding participation in the study

-Eligibility questions:

Are you over 18?

Would you be able to understand and speak in English for the interview?

Have you moved voluntarily to the UK?

When did you move to the UK?

Did you experience post-natal depression or any symptoms resembling this condition since being here?

When did you experience post-natal depression?

Are you still experiencing this and/or any other acute mental health distress?

*Note. Discuss definition of ‘postnatal depression’ used for this research if needed and appropriate: ‘Post natal depression’ is a term used to describe “depression suffered by a mother following childbirth, typically arising from the combination of hormonal changes, psychological adjustment to motherhood, and fatigue” (as stated by the Oxford dictionary”.*

*However, we also know that people have similar experiences, without necessarily using this term.*

-Further questions:

Do you have any additional needs or requirements that should be considered if you were to take part in the study?

-Summary and next steps:

I will send you an information sheet and consent form to review and sign, if you are happy to do this, we can then go ahead and book a time/date for the interview

Do you have any questions or concerns about the information sheet? Here is the consent form, are you ok to review and sign?

If consent form is signed and only once this is done, share: after the interview, you will receive a 20-pound voucher as a thank you for your time.

-Scheduling interview time and date: 1h; MS Teams; confidential space, approx. 7 questions; interested in hearing your experience, your understanding of PND even beyond a diagnosis or not.
