# Supplementary material for: Migrant mothers’ experiences of postnatal depression in the UK
Source: PLoS One. 2026 May 6;21(5):e0347584. doi: 10.1371/journal.pone.0347584 (PMC13148705; doi:10.1371/journal.pone.0347584)
Supplement: S3 File — (DOCX) [file pone.0347584.s003.docx]

Interview Guide

**Interview schedule (1h)**

Part 1:

-Hello, nice to meet you since our last call.

-This should last about 1 hour, please let me know if you need a break at any point. Should we plan this in now? What time? How long for?

-It is important that you are alone in the room.

-If at any point you change your mind please let me know and we can talk through this

-I will press record, as a reminder, these will be kept on a secure UH OneDrive and separate to any confidential information – transcripts will be anonymised.

-Any final questions or concerns before we start?

**Part 2: Interview questions**

*Intro:* The way I will ask you questions might feel a bit strange – I will not necessarily comment or reply to what you have said, in order to make sure I hear your full experience and interfere/influence this as little as possible. If you find yourself feeling distressed at any point, of course we will be able to take a pause to address this. If at any point, we need to move on to the next question to complete the interview in time, I will gently give you a nudge.

Essentially, I am really interested in hearing about your experience, and I might ask follow up questions to explore your answers further if needed.

I have 7 questions for you today; the first few will be solely about your experience moving to the UK and then I will ask more specific questions about your experience of post natal depression. How will you feel about this?

*General prompts: Can you say more about that? You used the word ‘…’, what does that mean for you? Can you give me an example?*

*General tips for interviewer: Do not respond like a clinician – no paraphrasing, no validation, summarising ect. except e.g. ‘I can tell this is really hard; would you like a pause’ if needed.*

1. **To start with, can you tell me about how you came to live in the UK?**

*Prompts: How did you come to decide moving to the UK? How do you feel about this decision?*

1. **What was it like for you, moving to the UK?**

*Prompts: What has it been like to leave your birth country? What challenges did you face? What benefits did you experience? How did you experience the change in culture/language? What was your support system like when you arrived? How does it feel to be a migrant in this country?*

*‘Post natal depression’ is a term used to describe “*depression suffered by a mother following childbirth, typically arising from the combination of hormonal changes, psychological adjustment to motherhood, and fatigue.” (as stated by the Oxford dictionary*”.*

*However, we also know that people have similar experiences, without necessarily using this term. I will now ask questions about your experience.*

1. **How do you experience the term ‘post-natal depression’ and when did you first notice things started to shift for you?**

*Prompts: When did you first notice that things became a bit more difficult? When did you notice this changing into post-natal depression difficulties? What was it like when you first noticed post-natal depression difficulties? What was your experience of navigating support for this? How did it feel to experience post-natal depression?*

1. **Tell me about the impact this experience had on you and how you responded to this?**

*Prompts: What would you say was most challenging about your experience? What would you say was most helpful throughout your experience?*

1. **How do you think your identity as a migrant (someone who moved to the UK) influenced your experience (of PND)?**

*Prompts: How is your identity as a migrant linked to your experience of PND?*

1. **Is there anything I have not asked about which you think is important to share regarding the topics we discussed today?**

*Prompts: Can you tell me more about that? Can you tell me about your experience of this?*

1. **Can you tell me what it was like for you, taking part in the interview today?**

*Prompts: What was it like for you, being asked these questions? Can you tell me about what it feels like to participate in this study?*

________________________________________________________________________

Distress protocol

**The researcher will intervene if the participant is:**

• Experiencing anxiety or distress during the interview. The participant will be asked if

they would like to take a break and if they wish for the audio-recorder to be switched

off.

• Continuing to show signs of upset. The participant will be asked if they would like the

interview to end and if they would like the researcher to support them to call

someone to spend time with them, such as a family member or friend.

• Unduly distressed. The researcher will remain with the participant until they are calm

and composed. The participant may then decide to continue with the interview or not.

The interview will be terminated if:

• The participant decides to terminate the interview.

• The participant decides to participate in the interview at another time or place.

• The researcher considers the levels of distress too high and the interview process as

not supportive.

**The researcher will, with the participant’s consent:**

• Discuss the potential support services available for them to access.

• Seek permission and ask if they would like a family member, friend or someone from

the local community to call them to offer support.

• Relevant contact details and places of emotional support will be provided to all

participants.

**The researcher will:**

• Use their professional duty of care and code of conduct accordingly, if there is high

risk of serious harm, the researcher will contact services such as, emergency

services, to support and keep the participant safe.

**Part 3: Debrief (signposting, next steps…)**

Thank you for taking part in the interview today.

There are services and support resources that you might find helpful on the information sheet

*List from information sheet*

Please do not hesitate to contact me if any questions or concerns come up following from today. I will send you this information via email along with your 20-pound voucher.
