# Supplementary material for: Migrant mothers’ experiences of postnatal depression in the UK
Source: PLoS One. 2026 May 6;21(5):e0347584. doi: 10.1371/journal.pone.0347584 (PMC13148705; doi:10.1371/journal.pone.0347584)
Supplement: Attachment — Submitted filename: Reviewers response-PONE-D-25-47137.docx [file pone.0347584.s004.docx]

**General comments-**

The authors have identified an important, but often neglected area in health overlapping with Migration, Pregnancy, and Mental Health. Methodologically, indeed it is a robust approach. The methodological description and explanation made in the manuscript is also appreciable.

However, as it is an area overlapping between Migration, Pregnancy, and Mental Health (PND), the study report must be more specific on those attributes. The phenomenon of interest (PND) needs to be established to distinguish between experiences of PND and general hardship of migrant mothers. At least the same need to be discussed within the manuscript.

Having a clear distinction between the experience of migrant mothers with or without PND is essential. If cultural displacement, isolation etc. are also experienced by migrant mothers without PND, then the phenomenology describes the experiences of migrant mothers (not only those having PND). This needs to be specifically discussed through introduction and discussion.

**Specific Comments-**

1. The objectives indicated exploration of the experiences of migrant mothers suffering from PND, which is in line with the methodology (IPA) applied here. The study also captured the interplay between migration-related factors and perinatal mental health. However, a “so what” component need to be integrated, in terms of the implication based on the data.
2. In methodology, further description is needed for-

How were the participants approached in Social Media?

Although sample size calculation is not too stringent in Qualitative research, and same is based on the type of research question and methodology adopted, there has to be a brief description, how this sample size (Seven) was pre-determined/ determined?

How the criteria, ‘Fluent in English’ were assessed in social media?

How were the participants with PND identified?

3. The study tool: should we call this Interview Guide, instead of Interview Schedule? It would be a good idea if the author could share the Interview guide, to understand the data collection plan and flow of the interview (if possible, also the anonymous interview transcript after removing indirect identifiers)
4. I disagree with this statement related to manual data analysis (line 167-68). Irrespective of the use of software, qualitative data needs deep immersion into the data. Software can help us to organize the analysis only, unless one is using an AI tool.
5. The authors have nicely presented the group experiential themes. However, it appeared that potential normal migrant emotions and PND experiences are overlapping. Although being an IPA study it is not mandatory to compare the experiences of migrant mothers having and not having PND, but a paragraph/ discussion on Distress (Common migration experience) and Disorder (PND) need to be incorporated in introduction/ discussion of this manuscript.
