# Supplementary material for: Migrant mothers’ experiences of postnatal depression in the UK
Source: PLoS One. 2026 May 6;21(5):e0347584. doi: 10.1371/journal.pone.0347584 (PMC13148705; doi:10.1371/journal.pone.0347584)
Supplement: Attachment — Submitted filename: Response to reviewers.docx [file pone.0347584.s006.docx]

**Point-by-point responses to reviewers’ comments regarding amendments**

We appreciate the time that was taken to review our paper and the thoughtful feedback that has been provided. Because I, as first author, led this research as part of my doctorate degree, guided by my co-author supervisors, I have taken the lead in clarifying/expanding on various aspects of the study as requested by reviewers, in collaboration with my co-authors

| **Comments from Reviewer 1:**  **Thank you for your feedback, please find the addressed comments below** | | | |
| --- | --- | --- | --- |
| **#** | **Comment** | **Response** | **Manuscript reference** |
| 1 | **Analysis**  The narrative is compelling, the themes are coherent, and the use of participant quotations is strong.  Limitations: The analysis does not sufficiently acknowledge how the researcher’s background, assumptions or interpretative lens shaped the analytic process. A reflexivity section is recommended, detailing: -researcher positionality -emotional and cognitive responses during analysis -potential biases -steps taken to mitigate interpretative influence | Thank you for your positive feedback about the findings.  I have expanded on my positionality (background, assumptions and interpretative lens) and added a reflexivity section to detail how this positionality shaped the analytic process. | Revised manuscript, pages 6-8 |
| **Comments from Reviewer 2**  **Thank you for your feedback, please find the addressed comments below** | | | |
| **#** | **Comment** | **Response** | **Manuscript reference** |
| 1 | If possible try to take more samples for reliable results. | Thank you for your suggestion. I am unable to take more samples as this research project has now ended. As this was a qualitative IPA study, 'dependability' rather than 'reliability' was aimed for in relation to the results, which relies less on sample size and more on "the extent to which the findings are consistent in relation to the contexts in which they were generated" (Frambach et al., 2013, p. 552).  A sentence has been added to provide context for the number of participants. | Revised manuscript, page 10 |
| 2 | Also addition of Gantt chart is advisable for qualitative studies. | I used a project timeline as per programme requirement to visually plan, schedule and track the different stages of the research project. This timeline included ethics approval process, sampling, recruitment, data collection and analysis. I have now included this timeline as a supplementary file. | Supplementary file ‘S1_Project Timeline’ |
| 3 | Minor corrections required in terms of sample size calculation and minimum sample size numbers. | Sample size calculations were not included in this study due to its qualitative nature and reliance on theoretical sampling rather than probability sampling (Lim, 2025).  Unlike quantitative studies, IPA determines data size based on  homogeneity, complexity and time constraints. Although the sample size is ‘small’, it was considered sufficient in terms of depth of findings (Clarke, 2010; Smith et al., 2022) | N/A |
| **Comments from Reviewer 3**  **Thank you for your feedback, please find the addressed comments below** | | | |
| **#** | **Comment** | **Response** | **Manuscript reference** |
| 1 | The objectives indicated exploration of the experiences of migrant mothers suffering from PND, which is in line with the methodology (IPA) applied here. The study also captured the interplay between migration-related factors and perinatal mental health. However, a “so what” component need to be integrated, in terms of the implication based on the data. | Implications section was expanded to address and integrate the ‘so what’ component of this study. | Revised manuscript, pages 29-30 |
| 2 | How were the participants approached in Social Media? | A recruitment poster was circulated on social media platforms and prospective participants could contact me directly to register their interest. Sentence added to describe this in Methodology section of the revised manuscript. | Revised manuscript, page 9 |
| 3 | Although sample size calculation is not too stringent in Qualitative research, and same is based on the type of research question and methodology adopted, there has to be a brief description, how this sample size (Seven) was pre-determined/ determined? | The estimated sample size (between 6-10) was based on methodological guidance by Smith et al. (2022). The final sample size of 7 was reached following several rounds of recruitment and with no further participants coming forward, thereby balancing methodological guidance with pragmatic considerations in the time constraints of the Doctorate.  Sentence added in manuscript to contextualise this. | Revised manuscript, page 9 |
| 4 | How the criteria, ‘Fluent in English’ were assessed in social media? | The criteria ‘fluent in English’ was assessed through:  -The prospective participants’ ability to read and understand the research flyer which included this criterion  -The prospective participants’ ability to register their interest in the study based on the above  -The prospective participants’ ability to understand, engage and respond during the pre-interview screening call  -The prospective participants’ ability to read, understand and ask questions about the information and consent forms  In line with the critical realist epistemology of this research, I included participants’ own judgment of whether they were fluent or not to be a contributing factor in my own judgment of their ability to meet this criterion.  I have added the “screening call guide” in the PLOS supplementary files. | Supplementary file “S2_Screening call guide” |
| 5 | How were the participants with PND identified? | Participants with PND were identified through:  -Research advertisement stating the criterion ‘self-diagnosed or diagnosed by medical professional’.  -Pre-screening call with questions around participant eligibility (see supplementary file “Screening call guide”).  Whilst a clinical diagnosis is one way of recognising PND, this is not the only ‘metric’ for experiencing of this phenomenon and in order to account for systemic bias, service waiting times and barriers of stigma, it was considered appropriate, in line with the critical realist epistemology and phenomenological nature of this research, to include participants’ own judgment of whether they identified as having experienced PND to be a contributing factor in my own judgment of their eligibility regarding this criterion.  Sentences have been added within the Methodology section to reflect this. | Revised manuscript, page 9  Supplementary file “S2_Screening call guide” |
| 6 | The study tool: should we call this Interview Guide, instead of Interview Schedule? It would be a good idea if the author could share the Interview guide, to understand the data collection plan and flow of the interview (if possible, also the anonymous interview transcript after removing indirect identifiers) | Thank you for this suggestion. Interview schedule has been amended to ‘Interview guide’.  I have added “Interview guide” as a supplementary file.  Even after removing indirect demographic identifiers, anonymous interview transcripts cannot be shared publicly because ethics approval was not granted for this. Participants did not provide consent to share their interview transcripts in full. Given the identifiability risks inherent with qualitative data and the sensitive nature of this topic, it was not deemed appropriate to build publicly sharing raw data into the study design and ethics approval and consent processes. | Revised manuscript, page 10  Supplementary file “S3_Interview guide” |
| 7 | I disagree with this statement related to manual data analysis (line 167-68). Irrespective of the use of software, qualitative data needs deep immersion into the data. Software can help us to organize the analysis only, unless one is using an AI tool. | Thank you for pointing this out. I understand where the wording of this may have inadvertently created direct comparison and agree with your point around this. I have now removed the comparison with the use of software. | Revised manuscript, page 11 |
| 8 | The authors have nicely presented the group experiential themes. However, it appeared that potential normal migrant emotions and PND experiences are overlapping. Although being an IPA study it is not mandatory to compare the experiences of migrant mothers having and not having PND, but a paragraph/ discussion on Distress (Common migration experience) and Disorder (PND) need to be incorporated in introduction/ discussion of this manuscript. | Migrant emotions and PND experiences are overlapping because the phenomenon being studies is “Migrant mothers’ experiences of PND” therefore implying that participants in this study do hold overlapping and intrinsically linked experiences of both migrant emotions and PND. I added a sentence to highlight this before the findings.  We added sentences to guide the reader through the concepts of distress and disorder in relation to ‘PND as experienced by migrant mothers’ throughout the introduction and discussion. | Revised manuscript, page 4 and page 24 |
